# Additively Manufactured Mechanically Tunable Cavity Resonator for Broadband Characterization of Liquid Permittivity

**DOI:** 10.3390/s25237145

**Published:** 2025-11-22

**Authors:** Thipamas Phakaew, Thet Pai Oo, Muhammad Uzair, Pruet Kowitwarangkul, Piyapat Chuchuay, Rungsima Yeetsorn, Danai Torrungrueng, Nonchanutt Chudpooti, Suramate Chalermwisutkul

**Affiliations:** 1 The Sirindhorn International Thai-German Graduate School of Engineering, King Mongkut’s University of Technology North Bangkok, Bangkok 10800, Thailand; thipamas.p@email.kmutnb.ac.th (T.P.); thetpai.oo@email.kmutnb.ac.th (T.P.O.); muhammad.uzair@email.kmutnb.ac.th (M.U.); pruet.k@tggs.kmutnb.ac.th (P.K.); piyapat.c@tggs.kmutnb.ac.th (P.C.); rungsima.y@tggs.kmutnb.ac.th (R.Y.); 2 Department of Teacher Training in Electrical Engineering, Faculty of Technical Education, King Mongkut’s University of Technology North Bangkok, Bangkok 10800, Thailand; danai.t@fte.kmutnb.ac.th; 3 Department of Industrial Physics and Medical Instrumentation, Faculty of Applied Science, King Mongkut’s University of Technology North Bangkok, Bangkok 10800, Thailand; nonchanutt.c@sci.kmutnb.ac.th

**Keywords:** tunable cavity resonator, additive manufacturing, dielectric constant, liquid sensing

## Abstract

This paper presents the design, fabrication, and experimental validation of a metal 3D-printed mechanically tunable cavity resonator operating in the hybrid TM–coaxial resonant mode for the broadband characterization of liquid permittivity. The proposed structure was developed based on a cylindrical cavity by incorporating a disc-terminated metallic tuning stub, which enables continuous frequency adjustment from 0.5 GHz to 3.0 GHz while maintaining a maximum unloaded Q-factor of 284 at 1 GHz under air-filled conditions. The tuning mechanism allows for precise frequency selection for characterizing materials exhibiting frequency-dependent permittivity. To demonstrate its sensing capability, the resonator was applied to characterize ethanol–water mixtures, where resonant frequency shifts were correlated with ethanol concentration at representative baseline frequencies of 1.00 GHz, 2.00 GHz, and 2.94 GHz. The sensor achieved frequency/dielectric constant resolutions of 0.39, 1.34, and 4.20 MHz and average concentration errors of 1.25%, 3.73%, and 2.49%, respectively. Moreover, polynomial fitting models enabled the accurate extraction of dielectric constants with an average deviation below 0.5% compared with a commercial dielectric probe system. The combination of frequency tunability, compact geometry, and compatibility with additive manufacturing establishes the proposed cavity resonator as a versatile platform for broadband dielectric spectroscopy, chemical sensing, and liquid characterization.

## 1. Introduction

The dielectric behaviour of materials, defined by their complex permittivity (ε=ε′−jε″), lies at the heart of modern electromagnetic sensing and material characterization. It determines how a material stores and dissipates electromagnetic energy, important for numerous industrial applications ranging from industrial process monitoring to the development of next-generation communication and sensing devices. Accurate permittivity data are indispensable for quality control in food and beverage production, such as moisture evaluation in honey [1] and alcohol grading in liquors [2,3]; the real-time detection of contaminants in dry foods [4]; lubricating oils [5]; water in oil [6]; and advanced dielectric substrates for high-speed printed circuit boards [7]. They also enable the optimization of catalytic and sorption media operating at temperatures exceeding 600 °C in exhaust gas after-treatment processes [8] and those exceeding 1500 °C in measuring the dielectric properties of microwave low-loss [9]. In these diverse applications, a sensing platform that is rapid, non-destructive, and broadband is crucial for capturing the frequency-dependent nature of dielectric dispersion. The microwave cavity perturbation technique fulfils most of these requirements by translating sub-percent variations in permittivity into measurable shifts in resonant frequency (Δ*f*) and the unloaded quality factor (Δ*Q*). Among such structures [10], the cylindrical TM_010_ cavity resonator stands out for its strongly confined axial electric field, simple analytical modelling, and compact geometry capable of maintaining a high Q-factor even in miniature configurations. However, its inherently narrow resonance bandwidth restricts its applicability to broadband characterization.

Significant progress has been achieved in the advancement of microwave cavity-based dielectric measurement techniques, particularly toward improving the accuracy and reliability of permittivity extraction for complex materials. The conventional first-order cavity perturbation theory provides satisfactory results when the sample volume is considerably smaller than the cavity volume, ensuring that the perturbation introduced by the sample remains minimal [11,12]. However, its validity diminishes when dielectric support tubes, air gaps, or high-loss liquids alter the internal electromagnetic field distribution, leading to increased measurement uncertainty. To mitigate these limitations, researchers have developed a range of refined analytical and numerical approaches. The Ritz–Galerkin full-wave formulations incorporate structural discontinuities such as sample holes and wall roughness, thereby enhancing modelling precision [13,14]. Similarly, the radial stratification model introduces field stratification to account for dielectric liners and sample holders [15], while field analysis techniques have been proposed for concentric and multilayer cavity geometries to improve the characterization of polar and lossy liquids [16,17]. These methods collectively achieve sub-1% accuracy in permittivity estimation and have substantially advanced the theoretical understanding of cavity–sample interactions. Nevertheless, these models are almost exclusively implemented in single-frequency resonators, which inherently limit broadband characterization.

To extend the measurement bandwidth, alternative cavity configurations such as multi-hole (dual-mode) [18] and coupled-resonant-plate structures [19] have been introduced. While these approaches enable access to multiple resonant modes within a single structure, they require large physical dimensions (typically exceeding 230 mm in diameter), precise coupling alignment, and complex calibration procedures. Consequently, their practical deployment in compact, field-portable, or liquid-phase sensing environments remains restricted. Despite notable advances in modelling accuracy and multi-mode design, the lack of a compact, frequency-agile, and high-Q resonator continues to constrain the broadband characterization of dielectric materials. This unresolved limitation provides the motivation for developing a mechanically tunable cavity resonator capable of continuous frequency sweeping without sacrificing sensitivity or Q-factor performance, thus bridging the gap between fixed-frequency precision and broadband dielectric spectroscopy.

A mechanically tunable cavity resonator capable of sweeping a broad frequency range without compromising its quality factor presents a compelling solution to the limitations of conventional fixed-frequency systems. Such a structure enables dielectric dispersion mapping, allowing both the real and imaginary components of frequency-dependent complex permittivity (*ε*′(*f*) and *ε*″(*f*)) to be characterized in a single experiment, an essential capability for polar liquids such as ethanol–water mixtures, whose dielectric loss peaks vary with concentration. Moreover, it facilitates mode optimization, permitting the selection of operating frequencies that maximize sensitivity (Δ*f*/Δ*ε*′) or measurement stability (high *Q*) for a given material. From a practical standpoint, a tunable cavity also offers instrumental simplification, as a single device can replace multiple fixed-frequency resonators, thereby reducing calibration effort, manufacturing complexity, and operational cost in industrial environments.

In response to these needs, this work proposes a mechanically tunable, capacitively loaded, coaxial cavity resonator operated in the hybrid TM–coaxial resonant mode for the broadband dielectric characterization of liquids. The proposed configuration integrates a disc-terminated metallic tuning stub, which enables continuous frequency adjustment from 0.5 GHz to 3.0 GHz while maintaining a maximum unloaded Q-factor of 284 at 2.94 GHz under air-filled conditions. This tunable mechanism allows for precise frequency control for materials exhibiting frequency-dependent permittivity, providing versatility unattainable in conventional single-frequency systems. The resonator’s capability was experimentally validated using ethanol–water mixtures, where the measured resonant frequency shifts exhibited excellent correlation with ethanol concentration with the cavity’s baseline frequencies of 1.00 GHz, 2.00 GHz, and 2.94 GHz. The sensor achieved frequency/relative permittivity resolutions of 0.39, 1.34, and 4.20 MHz and average concentration errors of 1.25%, 3.73%, and 2.49%, respectively. With its compact geometry, broad tunability, and compatibility with metal additive manufacturing, the proposed resonator represents a novel platform for the non-contact, broadband dielectric characterization of liquids that, to the best of our knowledge, has not been reported in the literature. The design features a through-hole passing through the tuning stub and cavity, preserving a radially symmetric field distribution that is particularly suitable for the continuous online monitoring of flowing liquids in a PTFE tube, with the added capability of precise frequency tuning to track frequency-dependent permittivity—capabilities unattainable with conventional single-frequency cavity designs. The proposed resonator represents a robust and industrially viable platform for broadband dielectric spectroscopy, chemical process monitoring, and liquid characterization across diverse applications.

The remainder of this paper is organized as follows. Section 2 presents the design principles, evolution, and fabrication of the proposed tunable cylindrical cavity, including details of the metal additive manufacturing process and precision machining of the tuning stub assembly. Section 3 describes the experimental setup employed for dielectric measurements and provides both the simulated and measured results across multiple tuning lengths to validate the cavity’s broadband performance. It also analyzes the frequency shifting behaviour in relation to dielectric dispersion, compares the proposed design with conventional single-frequency cavities, and discusses the trade-offs between sensitivity and signal clarity under various operating conditions. Section 4 extends the application of the system to the extraction of dielectric constants for different liquid samples across multiple frequencies, demonstrating its adaptability for broadband characterization. Finally, Section 5 concludes this paper and presents a comparative assessment of the proposed resonator against established microwave cavity perturbation techniques, highlighting its potential as a compact and versatile platform for industrial and scientific dielectric sensing applications.

## 2. Design and Fabrication of Frequency Tunable Cavity Resonator

This section presents the design and fabrication of a mechanically tunable cavity resonator for the broadband dielectric sensing of liquids. The proposed design enables frequency adjustment via a movable tuning stub, allowing for adaptation to materials with frequency-dependent dielectric properties. This section begins with the theoretical basis and equivalent circuit model of the cavity, followed by design calculations targeting a 3.00 GHz unloaded resonance. The tuning mechanism, including stub geometry and range, is detailed next, and this section concludes with the fabrication process and mechanical drawings showing component dimensions and the assembly layout.

### 2.1. Working Principle

**Figure 1:**
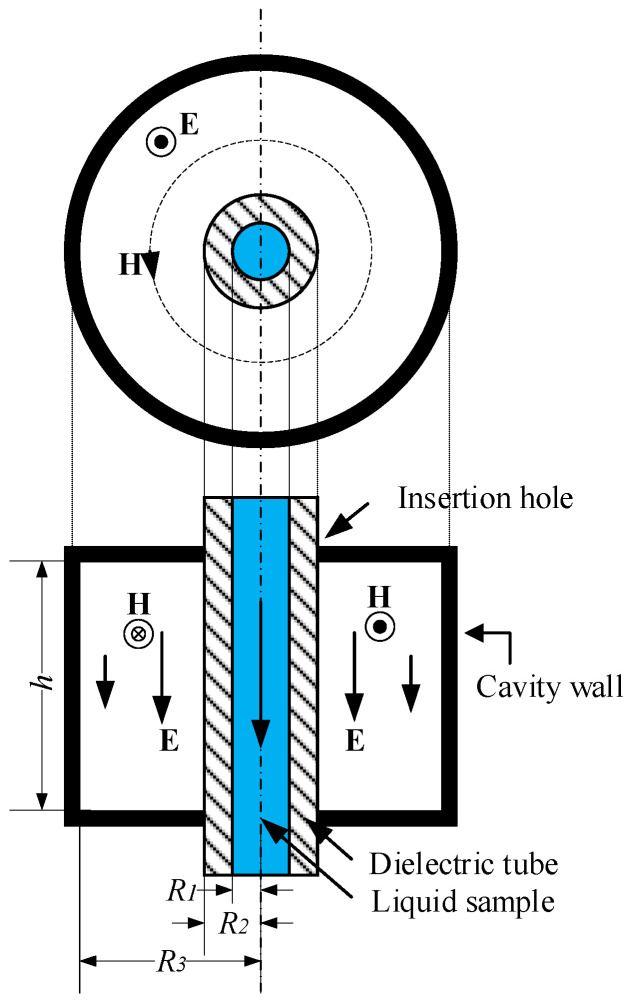
Fundamental configuration of conventional TM_010_ cylindrical cavity for measuring dielectric properties of liquid [16]. E denotes the electric field and H denotes the magnetic field.

In a previous study [16], the fundamental principle of using a TM_010_ cylindrical cavity for measuring the dielectric properties of liquids was based on introducing a small sample into the region of the strongest electric field within the cavity. This perturbation leads to a shift in the resonant frequency and a reduction in the quality factor (Q-factor) of the cavity resonator. To ensure minimal disruption to the electromagnetic field distribution, it is assumed that the volume of the sample defined by radius *R*_1_ (see Figure 1) is significantly smaller than the volume of the cavity and that its presence causes negligible disturbance to the cavity’s geometry and field structure. The fundamental configuration of the TM_010_ cylindrical cavity for measuring the dielectric properties of liquid is illustrated in Figure 1. It consists of a cylindrical cavity with an internal dielectric tube used to guide the liquid sample into the high-field region. The cavity has a radius *R*_3_ and height *h*, while the sample insertion hole has a radius *R*_2_, matching the outer diameter of the dielectric tube. This configuration ensures precise alignment and consistent electromagnetic coupling between the cavity and the liquid under test (LUT).

However, this conventional approach operates at a single fixed resonant frequency, which can be a limitation when characterizing materials whose dielectric properties vary strongly with frequency. If the permittivity of the LUT changes abruptly near the operating frequency due to material dispersion, accurate differentiation or validation becomes more difficult. To overcome this challenge, the present study proposes a mechanically tunable cavity resonator, which offers adjustable operating frequencies. This capability enhances measurement flexibility and improves adaptability for a wide range of liquid materials.

**Figure 2:**
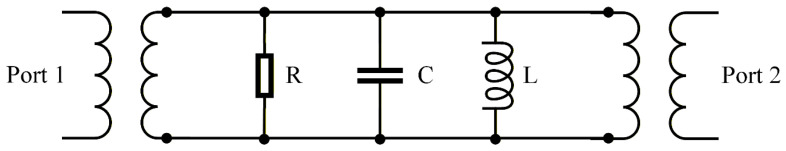
Equivalent circuit model of cavity resonator.

The equivalent circuit model of a two-port resonant cavity with magnetic coupling is represented by a parallel *LCR* resonant circuit, as shown in Figure 2 [20]. This model effectively captures the resonant behaviour of the cavity by simplifying it into lumped-element components including inductance *L*, capacitance *C*, and resistor *R* representing the dielectric loss of the material sample. The resonant frequency $f_{r}$ of the circuit is given by the following:

(1) $$f_{r}=\frac{1}{2\pi \sqrt{LC}}$$

This equation indicates that the resonant frequency is governed by the interplay between the inductive and capacitive elements of the cavity structure. In a physical cavity, these parameters are directly related to the geometric configuration and material properties of the resonator. Changes in either the effective capacitance (such as through loading with dielectric material) or inductance (through modifications of the magnetic path) will shift the resonance frequency. This simplified *LC* model provides a practical framework for analyzing and designing cylindrical cavity resonators for material characterization applications.

### 2.2. Design of Mechanically Tunable Cavity Resonator

An essential initial step in designing a TM_010_ cavity resonator for material sensing applications is to determine its resonant frequency in unloaded conditions, when the cavity is filled with air or vacuum and not yet loaded with any sample [20]. The resonant frequency is directly related to the inner cavity radius *R*_3_ (see Figure 1), as expressed in Equation (2), where *c* is the speed of light in vacuum, and $p_{01}$= 2.405 is the first root of the Bessel function *J*_0_, corresponding to the TM_010_ mode. For a target resonant frequency of 3.00 GHz, the radius *R*_3_ of the cavity can be calculated by rearranging Equation (2).

In practice, for the TM_010_ mode, cavity height *h* does not affect the resonant frequency. However, it is closely related to the quality factor *Q*, as shown in Equation (3), where the quality factor is inversely proportional to the cavity height. To use the cavity as an electromagnetic sensor, a high-quality factor is essential, as it enhances the accuracy in measuring the dielectric properties of samples. Therefore, to maximize *Q*, the cavity height is designed to be as short as practically possible, which in this case is 40 mm.

(2) $$f_{TM010}=\frac{c}{2\pi \sqrt{\mu_{r}\varepsilon_{r}}}\sqrt{{\left(\frac{p_{01}}{R_{3}}\right)}^{2}+{\left(\frac{l\pi }{h}\right)}^{2}}$$

(3) $$Q_{0}=\frac{\eta_{0}}{R_{s}}\frac{p_{01}}{2\left(\frac{R_{3}}{h}+1\right)}$$

**Figure 3:**
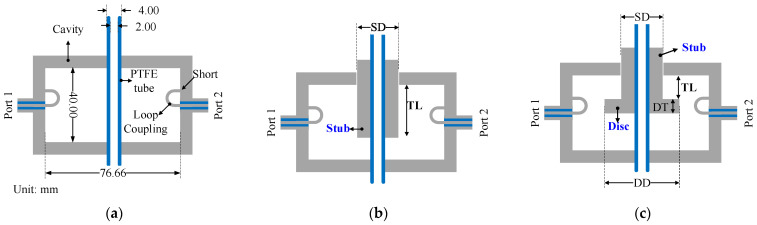
Evolution of tunable cavity designs: (**a**) Conventional fixed-frequency TM_010_ cavity [16]. (**b**) Mechanically tunable cavity with cylindrical stub. (**c**) Proposed design with cylindrical stub terminated by disc for enhanced frequency control.

The resonant frequency of a cavity can be tuned by modifying its effective capacitance or inductance. Figure 3 illustrates the evolution of cavity designs leading to the current implementation. The conventional fixed-frequency TM_010_ cavity (Figure 3a) offers no tunability, limiting its versatility for characterizing frequency-dispersive materials. To address this, a mechanically tunable design was introduced by inserting a cylindrical metallic stub with a diameter *SD* along the cavity axis ((Figure 3b), stub without disc), allowing for the control of the tuning length *TL*. However, this configuration exhibited a limited frequency tuning range. To enhance frequency agility and control, a planar disc with a diameter *DD* and a thickness *DT* was added to the end of the stub (Figure 3c). The proposed stub design with disc termination increases capacitive loading and thereby expands the frequency tuning range.

**Figure 4:**
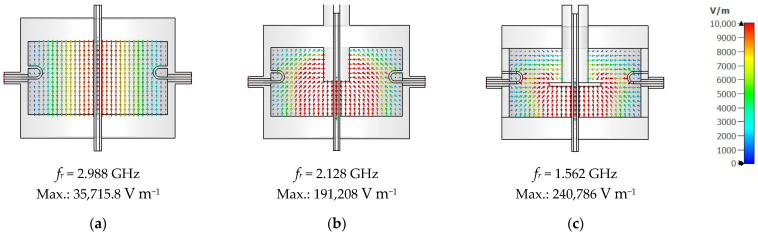
Simulated electric field distribution of three cavity configurations: (**a**) Conventional fixed-frequency TM_010_ cavity [16]. (**b**) Cavity with plain tuning stub. (**c**) Proposed design with tuning stub terminated by disc. The arrows indicate the instantaneous electric-field directions at a particular phase of the signal. Note that the modes in (**b**,**c**) are not pure TM_010_; but hybrid TM–coaxial resonant modes.

**Figure 5:**
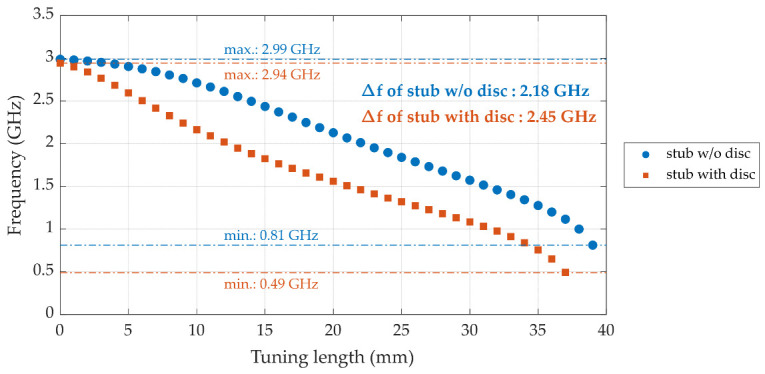
The simulated resonant frequency of the cavity featuring a tuning stub without disc termination (stub w/o disc) and a stub with disc termination (stub with disc) at various stub tuning lengths.

Figure 4 corroborates these findings by visualizing the simulated electric field distribution for each design variant. In the baseline cavity without a tuning element (Figure 4a), the field is radial-symmetrically confined along the axis, yielding the highest resonant frequency (*f_r_* = 2.99 GHz) with a modest peak field intensity (3.6 × 10^4^ V m^−1^). Introducing a plain cylindrical stub with *SD* = 15 mm (Figure 4b) lengthens the current path and adds series inductance, driving *f_r_* down to 2.13 GHz while simultaneously concentrating the electric field near the stub tip (peak 1.9 × 10^5^ V m^−1^). As a result, the cavity transitions from the original TM_010_ mode to a hybrid TM–coaxial resonant mode, in which the field distribution near the stub resembles a capacitively loaded coaxial section. When disc termination is added to the tuning stub (*SD* = 15 mm, *DD* = 30 mm, and *DT* = 2 mm; see Figure 4c), the electric field is further pulled toward the disc edges, markedly increasing local capacitive loading; this pushes *f_r_* down to 1.56 GHz and raises the peak field intensity to 2.4 × 10^5^ V m^−1^. The progressive shift in the field maximum from the cavity centre (Figure 4a) to the stub tip (Figure 4b) and finally onto the disc surface (Figure 4c) explains the larger tuning span achieved in the disc-terminated stub, as well as its enhanced resolution due to small axial adjustments. Thus, the field distribution analysis confirms that the disc-terminated stub not only broadens the tuning range but also provides stronger field confinement at the sensing region, improving both the frequency agility and dielectric sensitivity of the proposed cavity. As depicted in Figure 5, the proposed design achieved a broader tuning bandwidth from approximately 2.94 GHz down to 0.49 GHz compared to 2.99 GHz to 0.81 GHz for the stub w/o a disc, resulting in a total frequency range ∆*f* of 2.45 GHz and 2.18 GHz, respectively. The final configuration with *SD* = 15 mm, *DD* = 30 mm, and *DT* = 2 mm was optimized to achieve stable frequency shifts, maintain insertion loss within acceptable levels, and preserve relatively high unloaded Q-factors across the tuning range.

### 2.3. Tunable Cavity Resonator Fabrication

**Figure 6:**
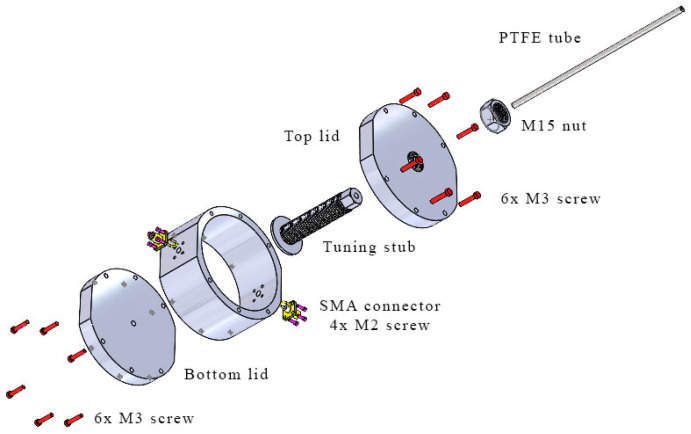
Exploded view of proposed tunable cavity resonator with all components.

**Figure 7:**
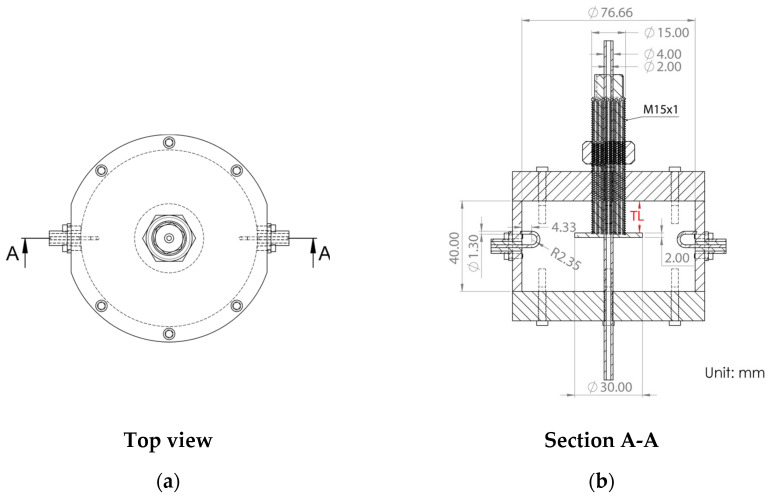
Detailed mechanical drawings of the proposed tunable cavity resonator with all dimensions in mm: (**a**) Top view. (**b**) Cross-sectional view (Section A–A).

The fabrication of the proposed tunable cavity resonator is illustrated in Figure 6, which presents an exploded view of all key components in the assembly. The top view of the fully assembled structure is shown in Figure 7a, while Figure 7b presents the cross-sectional view (Section A–A) with detailed dimensions. The assembly consists of a cavity body and bottom lid, both fabricated from stainless steel AISI 316L [21] using the metal additive manufacturing (AM) process, commonly referred to as metal 3D printing [22] via the MEAM (Material Extrusion Additive Manufacturing) process. The top lid, also made of stainless steel 316L fabricated using a Computer Numerical Control (CNC) machine, is designed for mechanical strength and long-term durability to resist wear from repeated tuning operations. The cavity has an inner radius of 38.83 mm and a height of 40 mm, which was selected to optimize the quality factor (*Q*) while maintaining a compact form. The cavity body and lids are assembled using M3 screws with a nominal outer diameter of 3 mm. The use of AM in this work serves as a proof of concept to demonstrate the feasibility of fabricating a tunable cavity by 3D printing. AM offers significant advantages for rapid prototyping, customized geometry realization, and efficient material use compared with conventional machining. It also enables quick design modification and re-fabrication for iterative optimization. In this study, the inner surface of the printed cavity was not post-processed; therefore, the roughness of the untreated 3D-printed surface contributes to a reduced quality factor compared with conventionally fabricated cavities. In addition, the limited electrical conductivity of stainless steel (approximately 1.35 × 10^6^ S/m) further lowers the Q-factor compared with aluminum (3.6 × 10^7^ S/m). These loss mechanisms were also incorporated into the simulation model to validate the measured results. Although the resulting Q-factor is lower than that of a conventionally machined cavity, it remains sufficient to evaluate the resonance shift caused by variations in the sample’s dielectric constant. Future improvements—such as surface polishing or electroplating to enhance the surface finish and increase the Q-factor, thereby improving measurement resolution and accuracy—will be investigated in future work.

Excitation is introduced using the coupling loop method, following the configuration described in [16], implemented through SMA connectors mounted on opposite sidewalls at a height of 20 mm from the bottom and secured with M2 screws. Each loop is formed using a 1.3 mm diameter copper wire, bent into a half-circle shape and connected directly to the centre conductor of the SMA connector, whereas the other end of the loop is connected to the ground and cylindrical body of the cavity.

**Figure 8:**
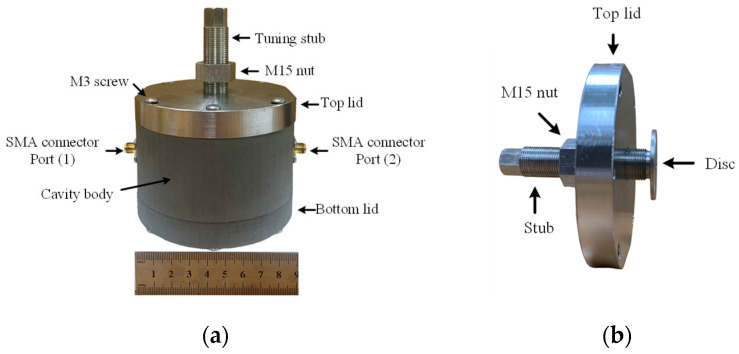
Photograph of fabricated tunable cavity resonator: (**a**) Full assembly. (**b**) Top lid with integrated tuning mechanism.

The tuning mechanism consists of a 1 mm threaded cylindrical stub with a shaft diameter of 15 mm, terminated with a disc measuring 30 mm in diameter and 2 mm in thickness. This tuning stub assembly is inserted from the top lid along the cavity’s central axis and is adjustable in the axial direction to vary the tuning length *TL* from 0 to 38 mm. An M15 stainless steel nut is threaded onto the tuning stub, serving both as a locking mechanism and a radiation choke to suppress electromagnetic leakage through the tuning port. Notably, the top lid, tuning stub, and M15 nut are all fabricated using precision turning processes on a CNC lathe rather than metal 3D printing, since the fine 1 mm thread pitch requires tight dimensional tolerances and surface finishing quality. A PTFE tube with an outer diameter of 4 mm is inserted vertically through a hole in the bottom lid to deliver the liquid under test (LUT) into the region of the strongest electric field. Figure 8 shows the fabricated components of the proposed tunable cavity resonator.

## 3. Results and Discussion

This section presents the experimental results and analysis of the dielectric characterization of ethanol–water mixtures using two complementary methods, by using a commercial dielectric measurement probe and the proposed tunable cavity resonator. The objective is to validate the cavity’s sensing capability by first benchmarking it against the standard dielectric measurements obtained using the commercial dielectric measurement probe and then demonstrating its tunability and sensitivity across different frequency ranges. Section 3.1 describes the preparation of the ethanol–water mixtures and their dielectric behaviour measured using the commercial probe. Section 3.2 then outlines the implementation and initial testing of the proposed tunable cavity under air-filled conditions, demonstrating its mechanical tuning mechanism and *Q*-factor stability. Finally, Section 3.3 presents the measurement results when the cavity is loaded with ethanol–water mixtures across a range of concentrations and tuning frequencies. The combined analysis of frequency shifts, resonance shape, and dispersion phenomena provides a comprehensive evaluation of the cavity’s performance and resolution as a broadband dielectric sensor.

### 3.1. Measurement of Liquid Samples’ Dielectric Property Using Commercial Probe

The ethanol–water mixtures used in this study were prepared by the Materials and Energy Applications Group (MfE) and Polymer Rubber Technology Group (PRT) at our faculty, using the standard dilution formula:

(4) $$C_{1}V_{1}=C_{2}V_{2}$$

where

*C*_1_ is the concentration of pure ethanol (100%).
*V*_1_ is the volume of pure ethanol.
*C*_2_ is the desired ethanol concentration (*E_c_*) (e.g., 90%, 80%, etc.).
*V*_2_ is the final volume of the prepared mixture (fixed at 100 mL).
Volume of distilled water = *V*_2_ − *V*_1_.

**Figure 9:**
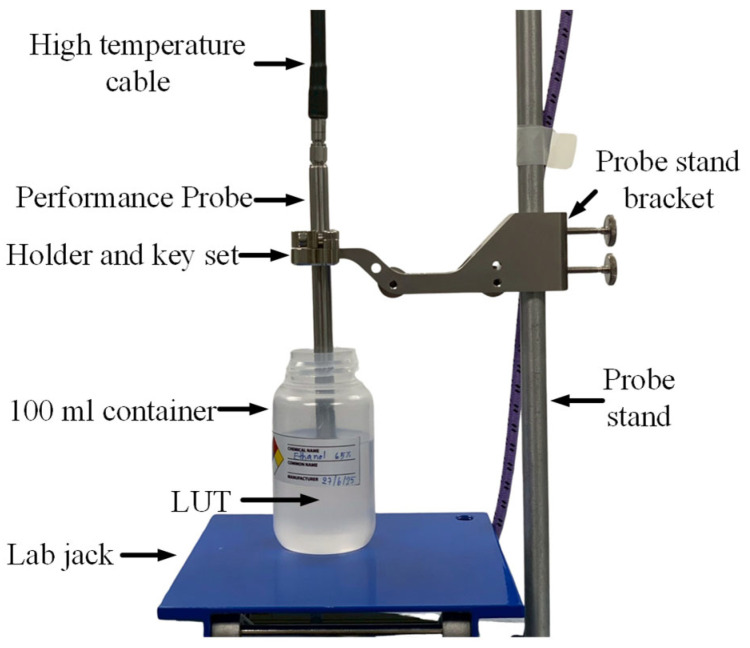
Dielectric property measurement setup using commercial dielectric probe system.

Figure 9 illustrates the experimental setup used for the dielectric characterization of liquid samples, specifically ethanol–water mixtures, using the commercial dielectric measurement probe. The probe is vertically mounted on a mechanical arm equipped with fine adjustment screws to ensure stable and repeatable immersion. The liquid under test (LUT) is placed in a 100 mL plastic container, which is positioned on a lab jack. This lab jack is used to gently raise the LUT to the probe, avoiding any disturbance to the fixed probe and ensuring consistent probe–sample contact. The probe is connected via a coaxial cable to an Agilent N5242A PNA-X network analyzer, with system control and data acquisition software of the N1500A Materials Measurement Suite. The measurement was configured to sweep over a frequency range of 500 MHz to 3.5 GHz, with 1201 measurement points and 16 average loops per point to enhance accuracy and reduce noise. All measurements were conducted within a class 10,000 cleanroom with a controlled room temperature of 23 °C to ensure reliable and repeatable dielectric property evaluation. To reduce noise and signal loss from coaxial cables and SMA connectors, a standard air–short–water (ID) calibration procedure with a water temperature of 23 °C was performed prior to measurement.

**Figure 10:**
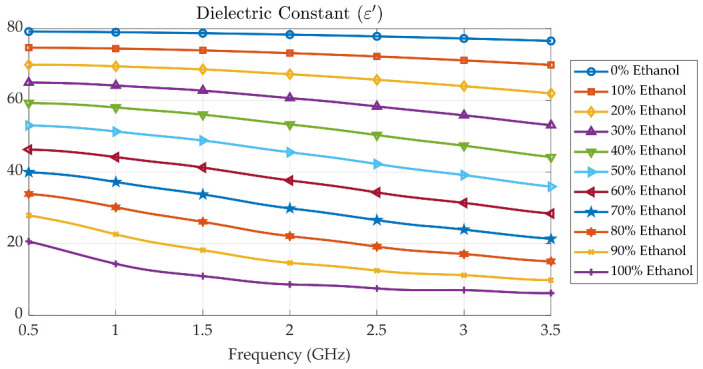
Measured dielectric constant *ε*′ of ethanol–water mixtures at various concentrations (0% to 100%) over frequency range of 500 MHz to 3.5 GHz.

**Figure 11:**
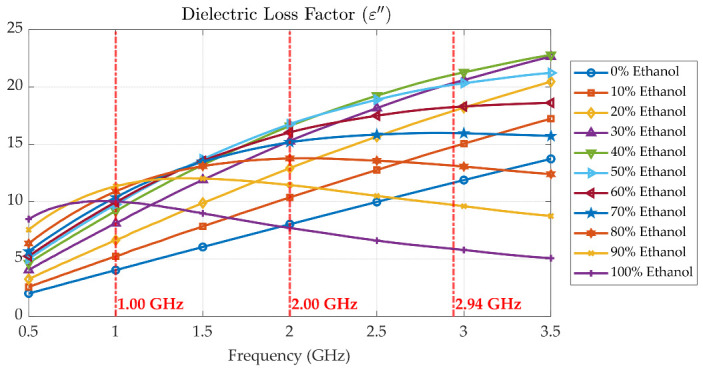
Measured dielectric loss *ε*″ of ethanol–water mixtures at various concentrations (0% to 100%) over frequency range of 500 MHz to 3.5 GHz.

**Figure 12:**
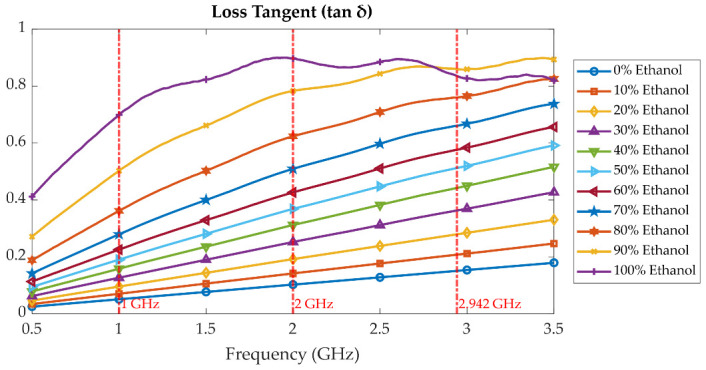
Measured loss tangent (tan δ) of ethanol–water mixtures at various concentrations (0% to 100%) over frequency range of 500 MHz to 3.5 GHz.

Figure 10, Figure 11 and Figure 12 present the measured dielectric constant *ε*′, dielectric loss *ε*″, and loss tangent tanδ of ethanol–water mixtures across concentrations from 0% to 100% over a frequency range of 500 MHz to 3.5 GHz. The dielectric constant *ε*′ shows a decreasing trend with both increasing frequency and ethanol concentration due to the lower permittivity of ethanol compared to water and the reduced dipole alignment at higher frequencies. The dielectric loss *ε*″, on the other hand, increases with frequency and exhibits a broad peak around 50% to 60% ethanol concentration, which is characteristic of dielectric dispersion due to molecular relaxation. This dispersion is attributed to complex hydrogen bonding and dipolar interactions between water and ethanol molecules, which create a heterogeneous environment that promotes enhanced energy absorption at intermediate concentrations. The strong dispersion observed in this range indicates the presence of a relaxation mechanism, where molecular dipoles can respond to alternating electric fields more effectively, resulting in higher dielectric loss. To illustrate this frequency-dependent behaviour within the tuning range of the proposed cavity, three representative baseline frequencies at 1.00 GHz, 2.00 GHz, and 2.94 GHz were selected. At 1.00 GHz, *ε*″ is relatively low across all concentrations, indicating minimal relaxation losses. At 2.00 GHz, the dispersion peak becomes more pronounced, especially near 50% ethanol, corresponding to the maximum dipolar loss. At 2.94 GHz, *ε*″ tends to decrease again, as dipoles can no longer reorient quickly enough at high frequency, resulting in reduced loss. This dispersion behaviour confirms the sensitivity of the probe system and supports the cavity’s ability to discriminate between liquid mixtures based on their relaxation dynamics.

### 3.2. Cavity Measurement Setup and Initial Results

**Figure 13:**
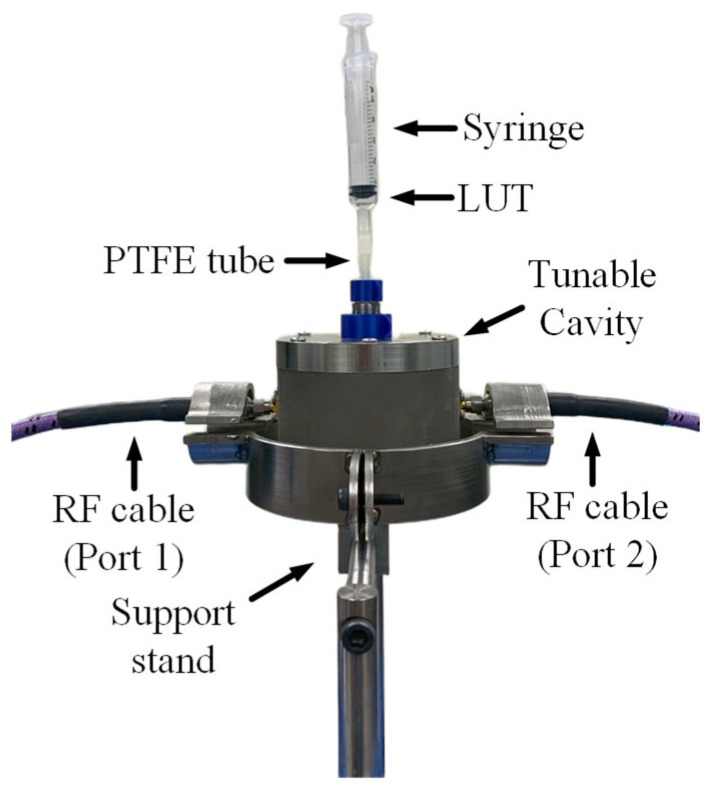
Measurement setup using proposed tunable cavity resonator.

Two-port measurements were conducted using an Agilent Technologies N5242A PNA-X network analyzer. To minimize measurement errors and signal loss introduced by the coaxial cables and SMA connectors, a standard open–short–load (OSL) calibration procedure was performed. The network analyzer was configured to operate over a frequency range of 500 MHz to 3.5 GHz, with a resolution of 20,001 measurement points. The measurement setup is illustrated in Figure 13. The proposed tunable cavity was placed on a support stand to allow a PTFE tube to pass through the lower lid. The input port was connected to port 1 of the PNA using an RF cable, while the output port was connected via another RF cable to port 2. The liquid under test (LUT) was introduced into the cavity through the PTFE tube using a syringe, ensuring a continuous liquid path free of air bubbles. The LUT’s temperature was maintained at a constant room temperature of 23 °C.

**Figure 14:**
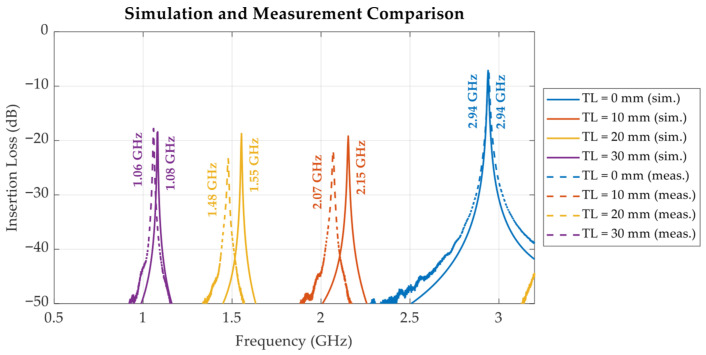
Simulated and measured insertion loss (S_21_) responses of proposed tunable cavity resonator under air-filled conditions at various tuning lengths *TL*.

Figure 14 illustrates the simulated and measured insertion loss (S_21_) responses of the proposed tunable cavity with air as the liquid under test (LUT), evaluated across various tuning lengths (*TL* = 0 mm, 10 mm, 20 mm, and 30 mm). The simulation was performed using CST Studio Suite, with the cavity modelled using stainless steel 316L (1.35 × 10^6^ S/m conductivity) with a surface roughness of 0.03 mm due to the metal 3D printing process [23] to accurately reflect the conductivity and loss characteristics of the fabricated prototype, and the *ε*′ and *ε*″ of the LUT measured using the commercial probe were used to model the liquid sample within the simulation. The results exhibit a clear and consistent downward shift in the baseline resonant frequency as the tuning length increases from 2.94 GHz at *TL* = 0 mm to approximately 1.06 GHz at *TL* = 30 mm. This frequency shift is attributed to the increased effective electrical length of the cavity, resulting from the insertion of a longer internal metallic stub. The corresponding Q-factors, summarized in Table 1, are 284.2 with *TL* = 0 mm, 218.8 with *TL* = 10 mm, 172.8 with *TL* = 20 mm, and 180.9 with *TL* = 30 mm. These values confirm that the proposed cavity maintains relatively high-Q performance across the tuning range, validating its effectiveness for tunable resonant applications.

**Figure 15:**
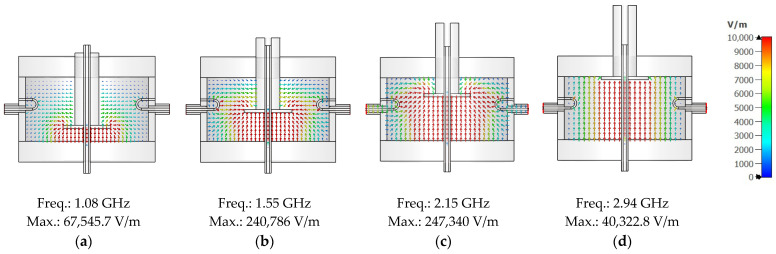
The simulated electric field distribution of the proposed tunable cavity resonator under the air-filled condition: (**a**) *TL* = 0 mm; (**b**) *TL* = 10 mm; (**c**) *TL* = 20 mm; (**d**) *TL* = 30 mm. The arrows indicate the instantaneous electric-field directions at a particular phase of the signal.

To further clarify the tuning mechanism, Figure 15 presents the simulated electric field distribution within the cavity at *TLs* of 0 mm, 10 mm, 20 mm, and 30 mm under air-filled conditions. As shown in Figure 15a, at *TL* = 0 mm, the electric field is primarily concentrated near the central region of the cavity. However, in Figure 15b through Figure 15d, corresponding to *TL* = 10 mm, 20 mm, and 30 mm, the field distribution progressively shifts toward the metallic disc. This shift results from the extended stub structure, which increases the effective path length within the cavity. Consequently, the resonant frequency decreases, as also observed in the S_21_ response. The field visualizations clearly demonstrate that mechanical tuning alters the cavity’s resonant behaviour by modifying its internal electromagnetic boundary conditions, thus enabling precise and effective frequency control.

The simulation and measurement results presented in Figure 14 show strong agreement in terms of resonant frequency trends and overall performance, thereby validating the accuracy of the proposed cavity model. The discrepancies observed—most notably for *TL* = 10 mm and 20 mm, where the magnitude and frequency of the |S_12_| peaks deviate considerably from simulation—cannot be explained by the tuner positioning tolerance of ±0.05 mm alone. Simulation studies show that the observed frequency shifts would correspond to stub positioning errors of approximately 1.2 mm for *TL* = 10 mm, 0.7 mm for *TL* = 20 mm, and 0.4 mm for *TL* = 30 mm, far exceeding the stub positioning tolerance of ±0.05 mm. The deviations between measurement and simulation can therefore be attributed to other practical factors, such as fabrication tolerances and unmodelled parasitic effects (e.g., threaded elements on the tuning stub and minor power leakage at the lid interfaces and stub threads). These variations are further explained by the electric field distributions illustrated in Figure 15. Specifically, the degree of mismatch between the simulation and measurement results appears to correlate with the electric field intensity inside the cavity. For instance, configurations with higher internal E-field concentration (e.g., *TL* = 10 mm) exhibit more pronounced deviations between simulation and measurement. Conversely, when the E-field is relatively low (e.g., *TL* = 0 mm), the discrepancy is minimal. This observation implies that higher field intensities increase the sensitivity of the system to imperfections, including lid interfaces and tuner stub threads. Consequently, the system becomes more sensitive to loss mechanisms under high-field conditions, amplifying measurement uncertainty. Overall, these findings emphasize the importance of accounting for electromagnetic field strength when evaluating simulation accuracy and mechanical tolerances in cavity resonator designs.

### 3.3. Ethanol Solution Characterization with Proposed Tunable Cavity Resonator

**Figure 16:**
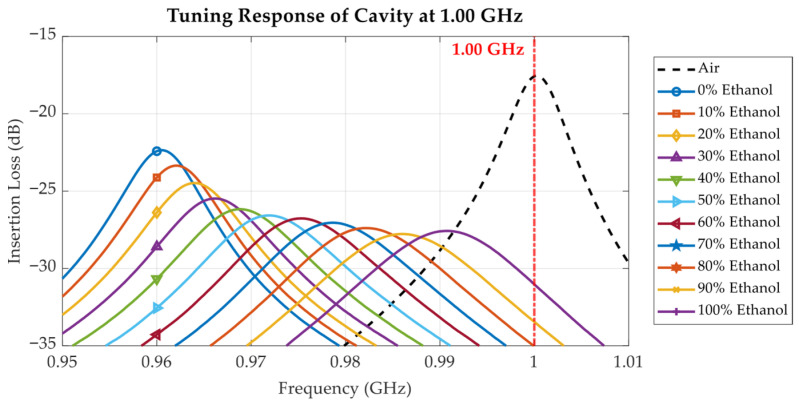
Measured insertion loss (S_21_) response for varying ethanol concentrations from 0% to 100%, with cavity tuned to 1.00 GHz.

**Figure 17:**
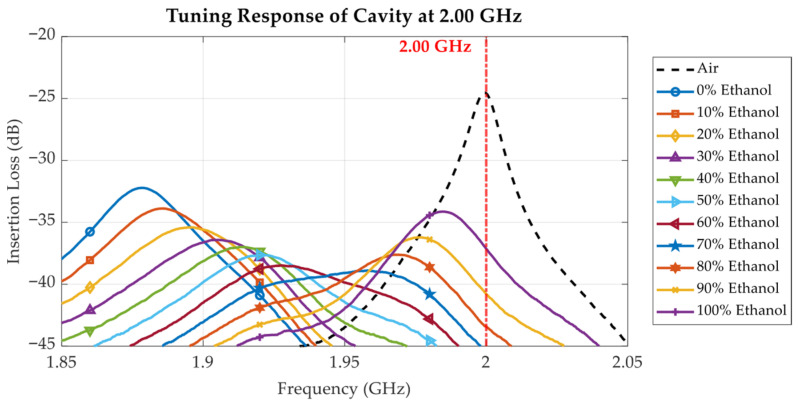
Measured insertion loss (S_21_) response for varying ethanol concentrations from 0% to 100%, with cavity tuned to 2.00 GHz.

**Figure 18:**
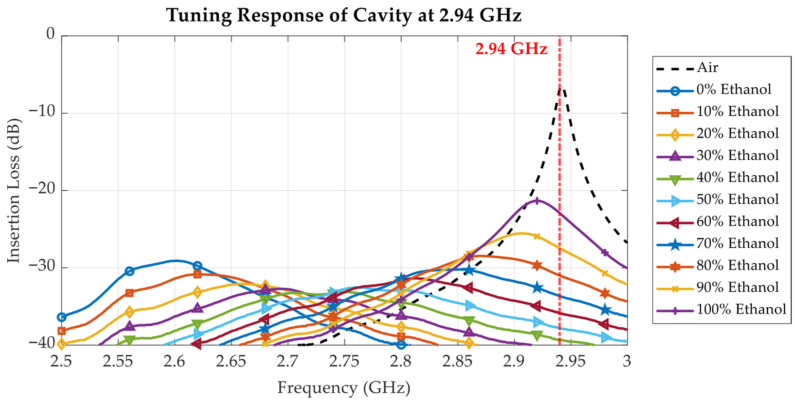
Measured insertion loss (S_21_) response for varying ethanol concentrations from 0% to 100%, with cavity tuned to 2.94 GHz.

**Figure 19:**
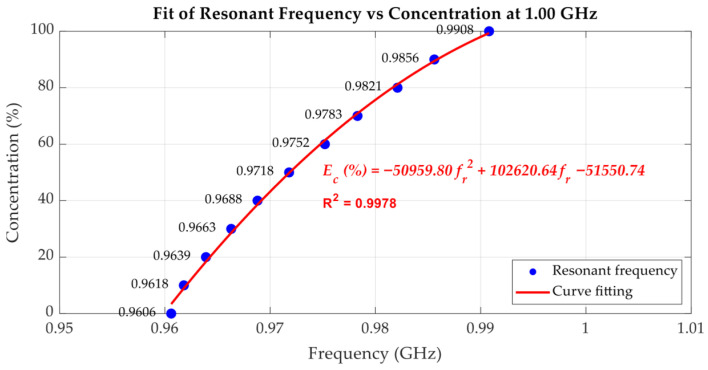
The second-order polynomial fitting of the resonant frequency used to predict the percentage of ethanol concentration, with the cavity tuned to 1.00 GHz.

**Figure 20:**
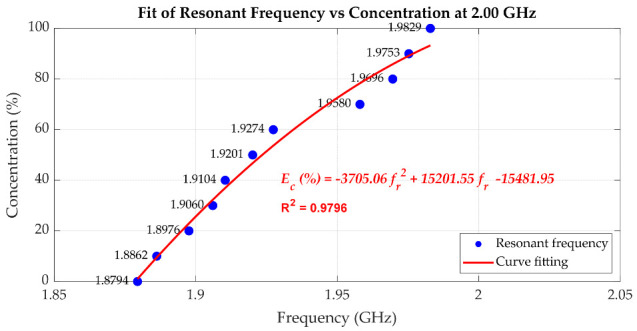
The second-order polynomial fitting of the resonant frequency used to predict the percentage of ethanol concentration, with the cavity tuned to 2.00 GHz.

**Figure 21:**
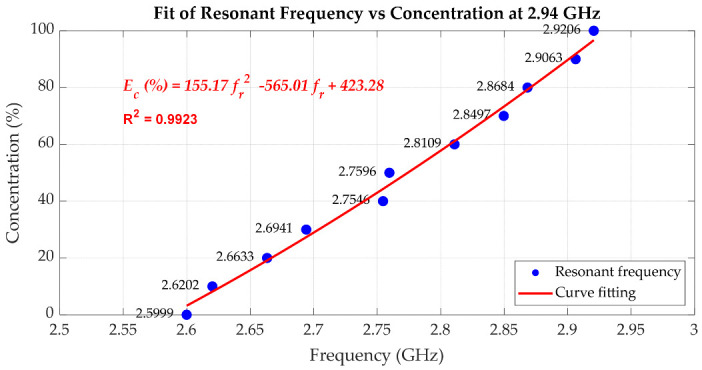
The second-order polynomial fitting of the resonant frequency used to predict the percentage of ethanol concentration, with the cavity tuned to 2.94 GHz.

To investigate the effects of the liquid under test (LUT) on the cavity’s resonance behaviour, the proposed tunable cavity was loaded with ethanol–water mixtures at varying ethanol concentrations ranging from 0% to 100%. Figure 16, Figure 17, Figure 18, Figure 19, Figure 20 and Figure 21 and Table 2, Table 3 and Table 4 show the measured results at three selected frequencies 1.00 GHz (low), 2.00 GHz (mid), and 2.94 GHz (high) which span the tunable range of the cavity. These frequencies were strategically chosen to evaluate the cavity’s sensitivity across a broad frequency spectrum and to assess its capability to detect dielectric variations at different resonant states.

In Figure 16, Figure 17 and Figure 18, the measured insertion loss (S_21_) of the loaded cavity is plotted for varying ethanol concentrations from 0% to 100%, with the unloaded cavity tuned to 1.00 GHz, 2.00 GHz, and 2.94 GHz, respectively. The results exhibit a systematic leftward shift in resonant frequency as ethanol concentration decreases. The resonant frequency shift directly correlates with the increased *ε*′ observed in Figure 10 as the ethanol concentration and frequency decreased. Since ethanol has a significantly lower permittivity compared to water, decreasing its proportion in the solution increases the dielectric loading of the cavity, thus reducing the resonance frequency. The resonant frequency shift is influenced not only by the *ε*′ of the sample but also by the baseline resonant frequency of the cavity. With the cavity tuned for higher baseline resonant frequencies (e.g., 2.94 GHz in Figure 18), the frequency shift per unit concentration change is more pronounced due to the larger LUT volume in the high-field region within the cavity. The sensor resolution can be computed using Equation (5). Considering the cavity loaded with air and DI water (0% ethanol), at 1 GHz, the dielectric constant changes from 1 to 79.00, resulting in a 30.2 MHz frequency shift; at 2 GHz, the dielectric constant changes from 1 to 78.33, resulting in a 103.5 MHz frequency shift; and at 2.94 GHz, the dielectric constant changes from 1 to 77.33, resulting in a 320.7 MHz frequency shift. Consequently, the resolutions of the cavity tuned at the 1 GHz, 2 GHz, and 2.94 GHz baselines are 0.39 MHz, 1.34 MHz, and 4.20 MHz, respectively. This results in greater sensitivity to dielectric variations at higher frequencies.

(5) $$\mathrm{sensor}\;\mathrm{resolution}\;=\;\frac{\Delta \mathrm{resonant}\;\mathrm{frequency}}{\Delta \mathrm{dielectric}\;\mathrm{constant}}$$

In addition to the frequency shift, the shape, depth, and sharpness of the resonance peaks vary significantly with both ethanol concentration and operating frequency. This behaviour is a direct consequence of the dielectric loss and dispersion effects illustrated in Figure 11. In ethanol–water solutions, the relaxation behaviour shifts with ethanol concentration. Solutions with low ethanol content (high water content) show no distinct relaxation within the measured frequency range up to 3.5 GHz, whereas at 40% ethanol, the dielectric loss begins to saturate near 3.5 GHz. With further increases in ethanol concentration, the relaxation peak appears at progressively lower frequencies, with pure ethanol exhibiting a relaxation frequency below 1 GHz. This trend occurs because ethanol is less polar than water. Increasing the water content enhances orientational polarization, allowing dipoles to follow higher-frequency fields and maintain a high permittivity over a broader frequency range. Conversely, as ethanol concentration increases, reduced molecular polarity and slower dipole relaxation shift the dielectric dispersion toward lower frequencies.

When the unloaded cavity is tuned to a baseline resonant frequency of 1 GHz (Figure 16), the dielectric loss *ε*″ remains relatively low across all ethanol concentrations (Figure 11), resulting in sharp resonance peaks and minimal signal attenuation. In this case, the resonant peak magnitude decreases monotonically as the ethanol concentration increases from 0% to 90%. As shown in Figure 11, at 2 GHz, the dielectric loss *ε*″ reaches its maximum for ethanol concentrations between 50% and 60%, corresponding to enhanced molecular relaxation. When the cavity’s baseline resonant frequency is tuned to 2 GHz, the measured insertion loss in Figure 17 reveals that the 50% and 60% ethanol solutions exhibit the lowest and broadest resonant peaks among all samples, indicating greater energy dissipation due to high dielectric losses. The resonant peak magnitude shows a non-monotonic dependence on ethanol concentration: it has a local maximum for pure ethanol (100%), decreases to a minimum at 50–60% ethanol, and then increases again, reaching another maximum for pure water (0%).

At 3 GHz, the dielectric loss *ε*″ reaches its maximum at approximately 50–60% ethanol concentration, as shown in Figure 11. When the cavity’s baseline resonant frequency is tuned to its highest value of 2.94 GHz, the measurement results for all LUT samples are as presented in Figure 18. In this case, the frequency shift caused by variations in ethanol concentration is more pronounced than those observed at baseline frequencies of 1 GHz and 2 GHz. However, the corresponding resonant peaks become broader and less distinct, a consequence of the higher dielectric loss at this frequency, as shown in Figure 11. Similarly to the 2 GHz case, the 50–60% ethanol solutions exhibit the lowest and broadest resonant peaks, and the variation in peak magnitude with concentration follows the same non-monotonic trend. The relationship between resonant frequency and ethanol concentration is established by examining the resonant frequency as a function of ethanol concentration.

Figure 19, Figure 20 and Figure 21 quantitatively illustrate this behaviour along with the corresponding second-order polynomial fits for three baseline frequencies (1.00 GHz, 2.00 GHz, and 2.94 GHz). In the case of the 1.00 GHz baseline frequency shown in Figure 19, the fitting equation is expressed as Equation (6) where $E_{c}(\%)$ denotes the ethanol concentration in percent, and $f_{r}$ represents the resonant frequency in GHz. To assess the uncertainty of the fitting equation, Table 2 presents the ethanol concentrations extracted from (6) compared with the actual ethanol concentrations and the corresponding absolute errors. The maximum error is 3.37% ethanol, while the average error is 1.25% ethanol. With the baseline frequencies of 2.00 GHz and 2.94 GHz, as shown in Figure 20 and Figure 21, respectively, the discontinuities of the resonant frequency trends, particularly near intermediate ethanol concentrations from 40% to 60%, reflect the impact of dispersion and molecular relaxation effects as previously characterized by both *ε*′ and *ε*″ in Figure 10 and Figure 11, respectively. In this concentration region, where relaxation losses are dominant, discontinuities in the concentration as a function of peak resonant frequency arise. The second-order polynomial fitting equation is expressed by Equation (7) for the 2.00 GHz baseline resonant frequency. Table 3 presents the ethanol concentrations extracted from (7) compared with the actual ethanol concentrations and the corresponding absolute errors, with a maximum error of 8.36% ethanol and an average error of 3.73% ethanol. At the baseline frequency of 2.94 GHz, the second-order polynomial fitting equation is expressed by (8). Table 4 presents the ethanol concentrations extracted from (8) compared with the actual ethanol concentrations and the corresponding absolute errors, showing a maximum error of 4.31% ethanol and an average error of 2.49% ethanol.

As mentioned before, with the cavity tuned at a higher baseline resonant frequency, the measurement resolution improves. However, due to the non-monotonic trend in *ε*″ regarding the concentration of the ethanol–water mixture at high frequencies, extracting the concentration using polynomial fitting results in relatively high concentration errors. To make use of the high resolution while avoiding a high concentration error, low and high ethanol concentration ranges can be separately fitted, e.g., from 0% to 60% and from 70% to 100% in the case of the 2 GHz baseline frequency. For the 3 GHz baseline frequency, the extraction of ethanol concentration around 40% to 50% is not practical due to the peaking dielectric loss and thus broad resonant peaks. However, the concentration ranges from 0% to 30% and from 60% to 100% can be separately fitted, yielding a lower concentration error compared to fitting over all concentration ranges. In conclusion, the cavity can be tuned at a higher resonant frequency if a high sensing resolution is desired by avoiding the characterization of samples with specific concentrations leading to peak dielectric loss. Conversely, at lower frequencies, although sensitivity is reduced, the cavity maintains a higher Q-factor and sharp resonance peaks, improving measurement clarity and robustness, particularly for lossy liquids.

(6) $$E_{c}(\%)=-50959.80f_{r}^{2}+102620.64f_{r}-51550.74;\;\;\;at\;1.00\;\mathrm{GHz}$$

(7) $$E_{c}(\%)=-3705.06f_{r}^{2}+15201.55f_{r}-15481.95;\;\;\;at\;2.00\;\mathrm{GHz}$$

(8) $$E_{c}(\%)=155.17f_{r}^{2}-565.01f_{r}+423.28;\;\;\;at\;2.94\;\mathrm{GHz}$$

## 4. Further Application

**Figure 22:**
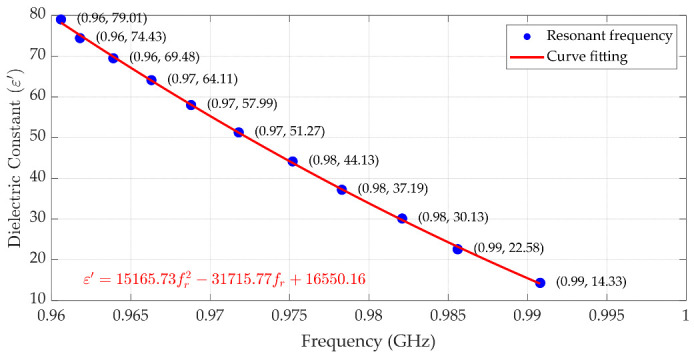
The second-order polynomial fitting of the resonant frequency used to predict the dielectric constant of liquid, with the cavity tuned to 1.00 GHz.

Beyond ethanol concentration sensing, the proposed cavity system can be extended to extract the dielectric constant of various liquids at different frequencies. This approach is based on the established relationship between the dielectric constant and the resonant frequency of the cavity loaded with LUT samples. Figure 22 illustrates the correlation between the dielectric constants of various ethanol concentrations and the measured resonant frequency using the cavity of which the baseline frequency is set to 1.00 GHz. This baseline frequency is selected since it is lower than the relaxation frequency of all LUT samples in our experiment. Unlike ethanol–water mixtures, if the LUT does not exhibit relaxation phenomena within the test frequency range, the trend in the dielectric constant as a function of resonant frequency is continuous.

A second-order polynomial fitting curve is applied to the measured data, providing a reliable mathematical model. The resulting fitting equation is expressed in (9). To validate the accuracy of this method, Table 5 compares the dielectric constants extracted using (9) with the values measured using the commercial probe. The comparison shows excellent agreement, with a maximum absolute error of 0.81 and an average error of 0.34. These results confirm that the proposed cavity-based approach provides an accurate and efficient method for dielectric constant determination across different frequencies, offering potential applications in liquid characterization over a broad frequency range.

(9) $$\varepsilon \prime =15165.73f_{r}^{2}-31715.77f_{r}+16550.16;\;\;\;at\;1.00\;\mathrm{GHz}$$

## 5. Conclusions

This work presents the development and experimental validation of a metal 3D-printed, mechanically tunable cavity resonator for wideband operation. With a disc-terminated tuning stub, the baseline resonant frequency of the cavity can be adjusted from 0.5 GHz to 3.0 GHz. The system was applied for ethanol concentration sensing, where resonant frequency shifts were correlated with ethanol concentration. Polynomial fitting models achieved average errors as low as 1.25% at a 1.00 GHz baseline resonant frequency, where the cavity also exhibited the highest Q-factor. However, at this frequency, the frequency shift to ethanol concentration resolution was relatively limited, making it more suitable for lookup-table applications with coarse concentration steps (e.g., 10% ethanol). At higher baseline frequencies (2.00 GHz and 2.94 GHz), dispersion and relaxation effects introduced less pronounced resonance for moderate ethanol concentration while offering high sensitivity. In particular, at 2.94 GHz, the cavity achieved a frequency resolution of 4.20 MHz per 1% ethanol concentration, enabling the finer discrimination of concentration variations and making it suitable for LUTs requiring higher resolution, such as 1% ethanol increments if the concentrations with high dielectric losses are skipped. An additional application was demonstrated in liquid dielectric constant extraction, where the proposed method achieved high accuracy with maximum deviations below 1% compared with commercial dielectric probe measurements.

Finally, when compared to the conventional microwave cavity perturbation methods reported in the literature, the proposed tunable cavity offers two major advantages: (i) frequency agility through geometric reconfiguration and (ii) the ability to achieve multi-frequency operation with a single compact cavity design. Table 6 presents a comparative analysis of previously reported TM_010_ cavity designs, highlighting the diversity of approaches in terms of operating frequency, physical size, quality factor, and application domain. For instance, studies such as [13,14] operated at a fixed frequency of 1.991 GHz using large cavities (Ø115.17 × 49.99 mm), achieving extremely high Q-factors in air (~15,890), but exhibited significant degradation in lossy media such as water (Q ≈ 430). Similarly, [15] introduced a radially stratified field model with a medium-sized cavity (Ø93.6 × 76.2 mm) to improve dielectric extraction, while [1] applied a compact (Ø92 × 40 mm) configuration to quantify water in honey, achieving moderate Q-values.

Multi-frequency operation was explored in [18] using a large-diameter (Ø230 mm) multi-hole cavity to extend the loss tangent measurement range, though the design was not optimized for compactness or tunability. In contrast, [16] employed the same physical cavity dimensions as this work (Ø77.66 × 40 mm), using a field-based technique to measure lossy liquids; however, its fixed frequency and reduced Q with water (~23.91) limited its resolution. High-frequency resonators in [5,6], operating at 5.344 GHz and 4.45 GHz, respectively, demonstrated effective sensing for oil contamination detection but required significantly different cavity sizes and showed sensitivity constraints in water or high-loss environments.

In comparison, our proposed cavity strikes a balance between compactness, tunability, and performance. With dimensions of Ø77.66 × 40 mm, it maintains a sufficient Q-factor (180–284 in air) across a broad tuning range without requiring multiple cavities. This tunable capability allows for the dielectric characterization of liquids with strong frequency dispersion or unknown properties, making it highly adaptable for chemical sensing, biomedical analysis, and industrial process monitoring.

## Figures and Tables

**Table 1:** Simulated and measured resonant frequency and Q-factor of proposed tunable cylindrical cavity resonator at various tuning lengths (*TL*) under air-filled conditions.

Tuning Length	Freq. (Sim)	Q-Factor (Sim.)	Freq. (Meas.)	Q-Factor (Meas.)
0 mm	2.94 GHz	489.83	2.94 GHz	284.20
10 mm	2.15 GHz	303.24	2.06 GHz	218.81
20 mm	1.55 GHz	304.51	1.33 GHz	172.81
30 mm	1.08 GHz	189.82	1.06 GHz	180.93

**Table 2:** A comparison of ethanol concentrations between the actual values and those extracted from Equation (6), with the cavity tuned to 1.00 GHz. The rightmost column shows the absolute error between the extracted and actual ethanol concentrations.

Resonant Frequency (GHz)	Ethanol Concentration (%)	Ethanol Concentration (%) Extracted from (6)	Absolute Error (% of Ethanol Concentration)
0.9606	0	3.37	3.37
0.9618	10	8.96	1.04
0.9639	20	18.38	1.62
0.9663	30	28.60	1.40
0.9688	40	38.62	1.38
0.9718	50	49.80	0.20
0.9752	60	61.37	1.37
0.9783	70	70.89	0.89
0.9821	80	81.22	1.22
0.9856	90	89.44	0.56
0.9908	100	99.34	0.66

**Table 3:** A comparison of ethanol concentrations between the actual values and those extracted from Equation (7), with the cavity tuned to 2.00 GHz. The rightmost column shows the absolute error between the extracted and actual ethanol concentrations.

Resonant Frequency (GHz)	Ethanol Concentration (%)	Ethanol Concentration (%) Extracted from (7)	Absolute Error (% of Ethanol Concentration)
1.8794	0	1.04	1.04
1.8862	10	9.53	0.47
1.8976	20	23.01	3.01
1.9060	30	32.33	2.33
1.9104	40	37.00	3.00
1.9201	50	46.79	3.21
1.9274	60	53.70	6.30
1.9580	70	78.36	8.36
1.9696	80	85.89	5.89
1.9753	90	89.23	0.77
1.9829	100	93.31	6.69

**Table 4:** A comparison of ethanol concentrations between the actual values and those extracted from Equation (8), with the cavity tuned to 2.94 GHz.

Resonant Frequency (GHz)	Ethanol Concentration (%)	Ethanol Concentration (%) Extracted from (8)	Absolute Error (% of Ethanol Concentration)
2.5999	0	3.18	3.18
2.6202	10	8.15	1.85
2.6633	20	19.14	0.86
2.6941	30	27.34	2.66
2.7546	40	44.31	4.31
2.7596	50	45.76	4.24
2.8109	60	61.12	1.12
2.8497	70	73.27	3.27
2.8684	80	79.30	0.70
2.9063	90	91.85	1.85
2.9206	100	96.70	3.30

**Table 5:** Comparison of dielectric constant of liquid between performance probe and those extracted from Equation (9), with cavity tuned to 1.00 GHz.

Resonant Frequency (GHz)	Dielectric Constant from Performance Probe	Dielectric Constant Extracted from (9)	Absolute Error Between Performance Probe and from (9)
0.9606	79.01	78.20	0.81
0.9618	74.43	75.13	0.71
0.9639	69.48	69.86	0.37
0.9663	64.11	63.99	0.11
0.9688	57.99	58.07	0.08
0.9718	51.27	51.22	0.06
0.9752	44.13	43.78	0.35
0.9783	37.19	37.30	0.12
0.9821	30.13	29.76	0.37
0.9856	22.58	23.20	0.62
0.9606	14.33	14.14	0.19

**Table 6:** Performance comparison of proposed tunable cavity resonator with previously reported cylindrical cavity resonator.

Ref.	Freq. (Air) (GHz)	Dimensions (mm)	Q-Factor (Air)	Q-Factor (Water)	Application
[13]	1.991	Ø115.17×49.99	15,890	430	Liquid permittivity with perturbation theory
[14]	1.991	Ø115.17×49.99	15,890	430	Analytical validation of liquid permittivity
[15]	2.46	Ø93.6×76.2	14,200	-	Extraction of complex εusing multilayer field model
[1]	2.4917	Ø92×40	2010.40	213.18	Honey moisture analysis
[18]	0.997/2.289 (TM_010_, TM_020_)	Ø230×19	4198/5797 (TM_010_, TM_020_)	-	Wide-range loss tangent characterization with multi-hole cavity
[16]	3	Ø77.66×40	445.58	23.912	Detection of wear particle contaminants in oil
[5]	5.344	Ø95×40	7517	-	Detection of contaminants in lubricating oil
[6]	4.45	Ø30×20	-	47 (Oil)	Measurement of water-in-oil concentration
This work	1.0–2.94	Ø77.66×40	180–284	23–115	Broadband dielectric characterization of liquids

## Data Availability

The data presented in this study are available on request from the corresponding author.
